# The Prevalence of Internet Gaming Disorder and Its Associated Factors Among University Students in Herat City, Afghanistan

**DOI:** 10.1002/puh2.70216

**Published:** 2026-04-03

**Authors:** Mohammad Shafi Saljuqi, Abdul Wahid Hamidi, Mohammad Masudi, Ali Rahimi, Shafiq Ahmad Joya, Zalmay Majidi, Nasar Ahmad Shayan

**Affiliations:** ^1^ Department of Curative Medicine Faculty of Medicine Jami University Herat Afghanistan; ^2^ Department of Pediatrics Faculty of Medicine Herat University Herat Afghanistan; ^3^ Department of Public Health and Infectious Diseases Faculty of Medicine Herat University Herat Afghanistan; ^4^ Department of Epidemiology and Biostatistics Schulich School of Medicine and Dentistry Western University London Ontario Canada

**Keywords:** internet addiction, problematic gaming, young adults

## Abstract

**Background:**

Internet gaming disorder (IGD) has become a growing concern among university students due to its potential impact on academic performance and mental health. This study aimed to assess the prevalence of IGD and its associated factors among university students in Herat City, Afghanistan.

**Methods:**

Data were collected using a structured questionnaire that included sociodemographic variables and the IGD‐20 Test. Participants were categorized using IGD‐20 thresholds: normal (20–49), at‐risk (50–69), and disordered (70–100). The Chi‐square test was applied to examine associations between sociodemographic characteristics and IGD levels, with statistical significance set at *p* < 0.05.

**Findings:**

The study revealed that 22.7% of participants were classified as disordered gamers, 50.4% as at‐risk gamers, and 27.0% as normal gamers. Single students had 50.6% at‐risk and 25.1% disordered. In contrast, married students had 49.6% and 14.7% (*p* = 0.009), and students whose mothers were illiterate showed 51.4% at risk and 18.9% disordered, compared with 54.5% at risk and 23.8% disordered for those with mothers holding a school degree and 38.2% at risk and 32.6% disordered for bachelor's or higher (*p* = 0.017).

**Conclusion:**

IGD is highly prevalent among university students in Herat, with more than half of the participants at risk or already classified as disordered gamers. These findings highlight the need for preventive interventions, awareness programs, and support strategies to mitigate the impact of IGD on students’ academic and social well‐being.

## Graphical abstract

Among 564 university students in Herat, internet gaming disorder was common: 27.0% normal, 50.4% at‐risk, and 22.7% disordered. IGD was higher in single students and showed a mixed association with mothers’ education; findings support targeted campus prevention and support.

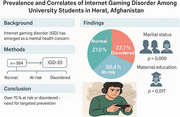

## 1 Introduction

Internet gaming has become one of the most widespread leisure activities worldwide, particularly in the form of massively multiplayer online role‐playing games (MMORPGs). These games provide entertainment, achievement, and social interaction, integrating deeply into contemporary digital culture [1, 2]. The growth of internet connectivity has further enhanced the social dimension of gaming, enabling players to interact across borders and creating a global gaming community. However, the rapid expansion of digital media has raised concerns about the potential addictive nature of gaming and its adverse effects on social, academic, and psychological well‐being [3].

Gaming disorder, as defined by the 11th Revision of the International Classification of Diseases (ICD‐11), is characterized by a persistent or recurrent pattern of gaming behavior marked by impaired control, prioritization of gaming over other activities, and continuation despite negative consequences, leading to significant functional impairment or distress [4, 5]. Similarly, the Diagnostic and Statistical Manual of Mental Disorders, Fifth Edition (DSM‐5) describes internet gaming disorder (IGD) as a condition warranting further study, identified by five or more of nine diagnostic criteria, such as preoccupation, withdrawal, tolerance, and unsuccessful attempts to control gaming behavior [3, 4]. Neuroimaging studies, including functional magnetic resonance imaging (fMRI), have demonstrated alterations in neural reward circuits in individuals with IGD that mirror those seen in substance use disorders, highlighting its potential neurobiological impact and the need for clinical attention [6, 7]. In addition to these neurobiological mechanisms, emerging evidence highlights the role of cognitive psychopathology in IGD, including maladaptive beliefs, persistent preoccupation with gaming, and dysfunctional thought patterns that reinforce compulsive gaming behavior and impair self‐regulation [8].

Globally, IGD affects approximately 3% of the population, with prevalence varying by region and measurement methods [9]. In Saudi Arabia, 21.5% of respondents in one study met the criteria for IGD. In Iran, a study showed that 3.7% of respondents met the criteria for IGD, whereas 2.1% of students at Tabriz University exhibited signs of the disorder [10, 11]. Countries such as China, Korea, and Taiwan consider gaming‐related problems significant public health concerns [12].

University students are particularly vulnerable due to academic pressure, social transitions, and limited mental‐health support [13, 14, 15]. Recent multinational and regional studies among university students and young adults have demonstrated strong associations between gaming disorder, psychological distress, impaired quality of life, and adverse functional and academic outcomes [16, 17, 18]. In Afghanistan—especially in Herat—the rapid expansion of internet access and smartphone use [19] has transformed leisure behaviors, making online gaming a dominant form of socialization among youth. Yet, to our knowledge, no peer‐reviewed study has assessed the prevalence or correlates of IGD in this context.

Given its potential to impair academic performance, mental health, and social functioning, IGD represents an emerging public health challenge. Therefore, this study was conducted among students at Herat University and Jami University to determine the prevalence of IGD and identify associated sociodemographic factors, thereby providing baseline evidence to inform prevention strategies and mental‐health interventions in Afghanistan.

## 2 Methods

### 2.1 Study Design and Population

This cross‐sectional study was conducted among students at Herat University and Jami University in Herat, Afghanistan, between October and November 2024. A stratified random sampling approach was employed to ensure representative participation across both universities. The strata were defined based on faculty (department) and class year to capture academic diversity. Within each stratum, proportional random sampling was applied on the basis of the total number of enrolled students, ensuring that larger faculties contributed proportionally more participants. Eligible participants were full‐time undergraduate students enrolled during the study period. Students who declined consent or submitted incomplete questionnaires were excluded from the analysis. Due to prevailing educational restrictions, only male undergraduate students were eligible for inclusion.

### 2.2 Sample Size

The sample size was determined using the following formula:

$$n=\frac{z^{2}p\left(1-p\right)}{e^{2}}$$

where *n* represents the sample size, *z* is the critical value for a 95% confidence interval (1.96), *p* is the estimated proportion of the population with the characteristic of interest (assumed to be 0.05 due to unknown prevalence), and *e* denotes the margin of error (0.04). Adjustments were made for the finite population of 7608 students (5882 from Herat University and 1726 from Jami University). The minimum required sample size was calculated to be 557 participants; after rounding, the final sample comprised 564 students, ensuring it accurately reflected the demographic distribution of the broader student population at both institutions.

### 2.3 Instrument

Data were gathered using a self‐administered structured questionnaire divided into three sections. The first section captured sociodemographic information, including gender, age, residence, living arrangements, field of study, parental education, family type, income level, and marital status.

The second section employed the IGD‐20 Test—a 20‐item instrument designed to assess IGD according to DSM‐5 criteria. Conceptualized and validated by Pontes et al. in English and also validated by Vahidi et al. in Persian [20]. On the basis of the total IGD score, participants in the current study were categorized as follows: Normal gaming (20–49) with minimal or no IGD symptoms, at‐risk gaming (50–69) with moderate symptoms that do not fully meet DSM‐5 criteria, and disordered gaming (70–100) with severe symptoms with significant impairment.

First, a pilot study with 20 students (10 from Herat University and 10 from Jami University), selected via convenience sampling, was conducted. Their responses were used solely for validation and excluded from the main analysis, yielding a Cronbach's alpha of 0.876. The third section of the questionnaire collected data on gaming behaviors, specifically focusing on the most frequently played and preferred online games among participants.

### 2.4 Data Analysis

Data analysis was performed using IBM SPSS Statistics version 27. Descriptive statistics (frequencies, percentages, means, and standard deviations where appropriate) were calculated to summarize participants’ sociodemographic characteristics and gaming behaviors. To examine the relationships between categorical variables and IGD classification, the Chi‐square test of independence was applied. Statistical significance was set at *p* < 0.05.

### 2.5 Ethics Approval and Consent to Participate

The study protocol was reviewed and approved by the Institutional Review Boards of Jami University (J.2024.1.27.6) and Herat University (250213‐18). All participants provided written informed consent before participating in the study. The confidentiality and privacy of participants were protected throughout the study in accordance with the Declaration of Helsinki and ethical guidelines for research involving human subjects.

## 3 Results

Participants were categorized using IGD‐20 thresholds: normal (20–49), at‐risk (50–69), and disordered (70–100). Table 1 shows sociodemographic characteristics. Most participants were aged 22–24 years (57.8%) and were predominantly single (77.1%). A slightly higher proportion belonged to extended families (52.1%), and most resided in urban areas (76.1%), with the majority living at home (74.1%). The highest representation was from Herat University (76.4%), and most participants were in their third year or above (42.2%). A significant proportion of students were from nonmedical faculties (75.0%). Regarding parental education, 38.8% of fathers had a bachelor's degree or higher, whereas 50.7% of mothers were illiterate. Regarding economic status, most participants reported a medium family income (50.0%).

**TABLE 1:** Sociodemographic characteristics of participants.

Variable		*N* (%)
Age categories	18–21	155 (27.5%)
	22–24	326 (57.8%)
	≥25	83 (14.7%)
Marital status	Single	435 (77.1%)
	Married	129 (22.9%)
Type of family	Nuclear family	270 (47.9%)
	Extended	294 (52.1%)
Living area	Urban	429 (76.1%)
	Rural	135 (23.9%)
Living place	Home	418 (74.1%)
	Dormitory	146 (25.9%)
University	Herat University	431 (76.4%)
	Jami University	133 (23.6%)
Year of study	First year	179 (31.7%)
	Second year	147 (26.1%)
	Third and above	238 (42.2%)
Faculty	Nonmedical	423 (75.0%)
	Medical	141 (25.0%)
Father's education	Illiterate	152 (27.0%)
	School degree	193 (34.2%)
	Bachelor and higher	219 (38.8%)
Mother's education	Illiterate	286 (50.7%)
	School degree	189 (33.5%)
	Bachelor's degree or higher	89 (15.8%)
Family income	High	166 (29.4%)
	Medium	282 (50.0%)
	Low	116 (20.6%)

**FIGURE 1:**
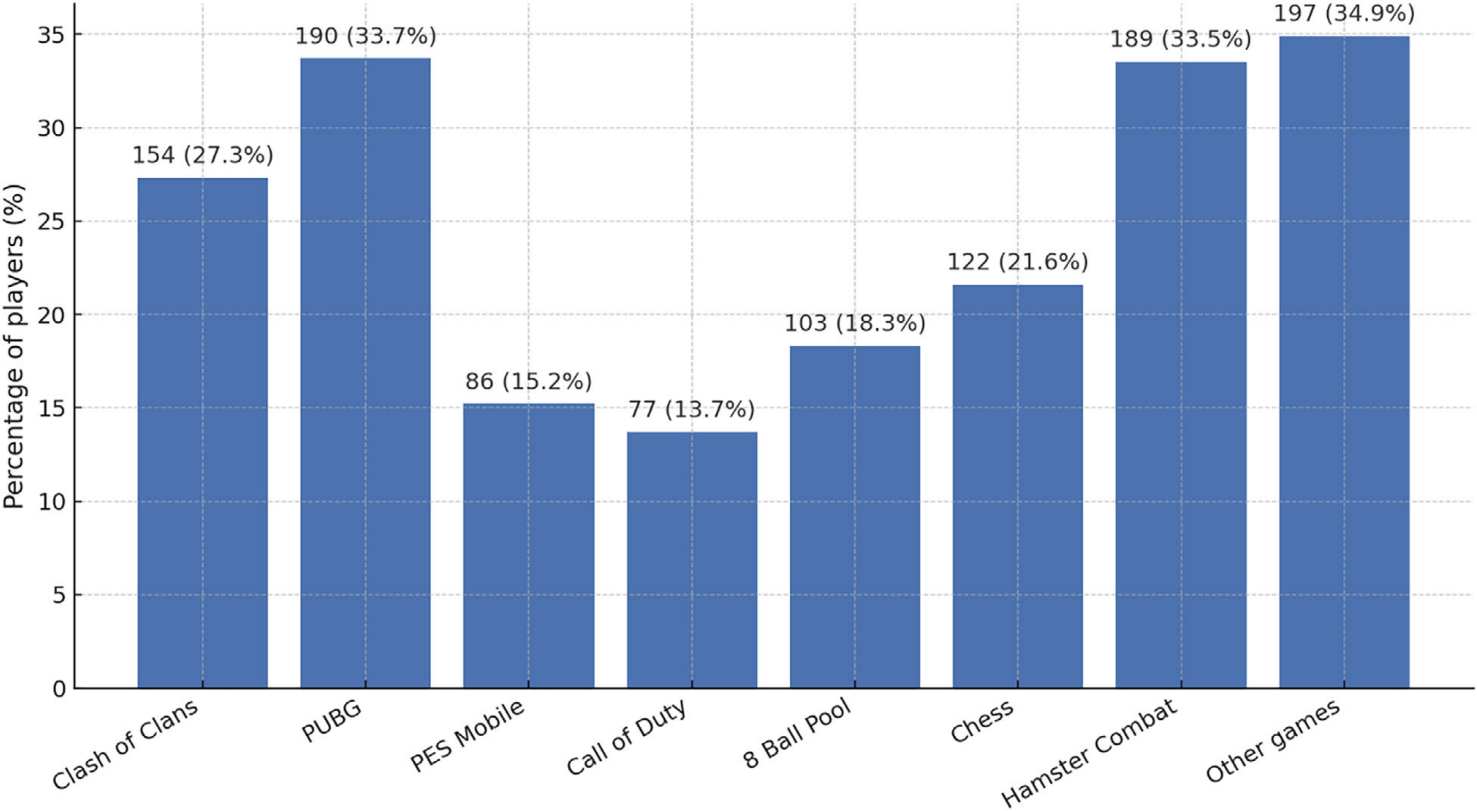
Distribution of game types played among participants.

Figure 1 shows types of games. Of the 564 participants, 34.9% reported playing games not specifically listed. PUBG was the most frequently played game, reported by 33.7% of participants, followed closely by Hamster Combat (33.5%). Clash of Clans was also a popular choice, with 27.3% of participants reporting gameplay. Chess, a strategic and intellectual game, was played by 21.6% of respondents, whereas 8 Ball Pool, a virtual billiards game, was played by 18.3% of respondents. PES Mobile, a football simulation game, was less commonly played, with 15.2% of participants engaging in it. Call of Duty, a popular first‐person shooter game, had the lowest player participation, with 13.7% of respondents reporting that they played it. These findings highlight the diverse gaming preferences among the participants, with a notable inclination toward multiplayer and strategy‐based games.

**FIGURE 2:**
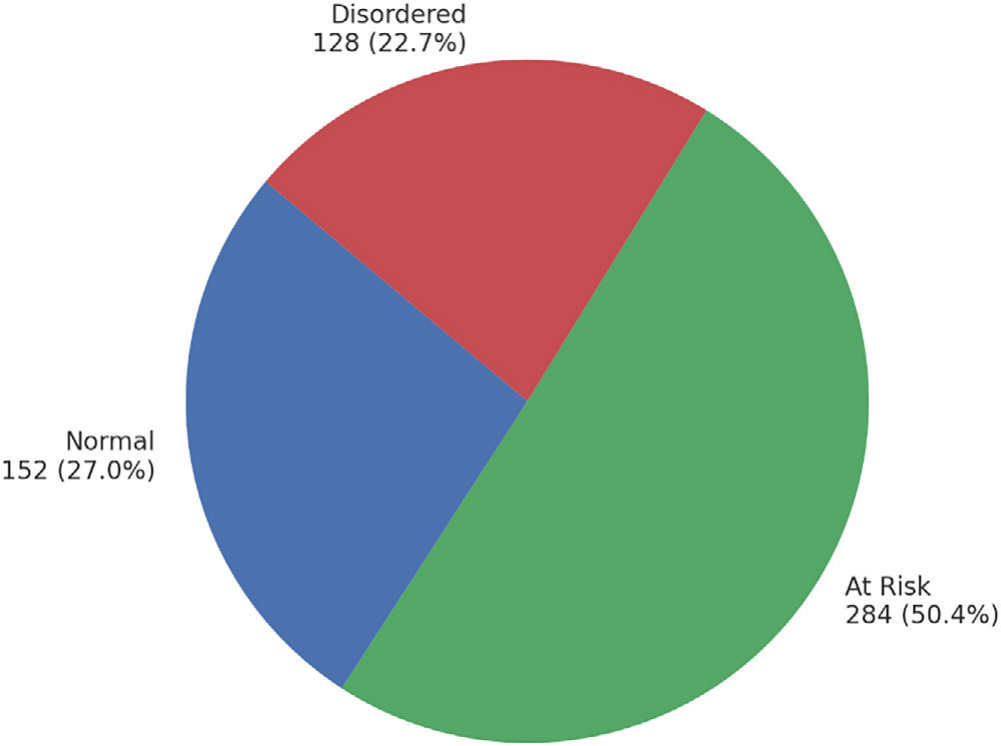
IGD categorization among participants.

Figure 2 shows IGD Categorization among Participants. On the basis of IGD‐20 score thresholds, 27.0% of participants were classified as normal gamers, whereas 50.4% were identified as at risk for IGD. Additionally, 22.7% of participants were categorized as disordered gamers, indicating a significant proportion of individuals exhibiting problematic gaming behaviors.

Table 2 shows associations among sociodemographic factors with IGD levels. Single students had 50.6% at risk and 25.1% disordered, versus 49.6% and 14.7% among married students (*p* = 0.009). This may reflect greater leisure time, fewer familial responsibilities, and higher levels of social isolation among single students, which can increase engagement in online gaming. By maternal education, students whose mothers were illiterate showed 51.4% at risk and 18.9% disordered, compared with 54.5% at risk and 23.8% disordered for those with mothers holding a school degree, and 38.2% at risk and 32.6% disordered for bachelor's or higher (*p* = 0.017). This relationship may reflect differences in parental awareness, guidance, and monitoring of digital behaviors, suggesting that higher maternal education could serve as a protective factor against problematic gaming. These findings suggest that marital status and maternal education may play meaningful roles in shaping gaming behaviors among university students. The overall prevalence of at‐risk (50.4%) and disordered gaming (22.7%) in this study appears considerably higher than the global average of approximately 3%–5% reported in meta‐analyses and neighboring countries such as Iran (2%–4%), China (5%–10%), and Saudi Arabia (21.5%). This discrepancy may indicate a local concern regarding digital engagement among youth, possibly influenced by limited recreational alternatives and post‐conflict social dynamics in Herat.

**TABLE 2:** Association between sociodemographic factors and IGD levels.

Variables		IGD categorizations			*p* values
		Normal	At risk	Disordered gaming	
		*N* (%)	*N* (%)	*N* (%)	
Age categories	18–21	39 (25.2%)	68 (43.9%)	48 (31.0%)	0.061
	22–24	89 (27.3%)	175 (53.7%)	62 (19.0%)	
	≥25	24 (28.9%)	41 (49.4%)	18 (21.7%)	
Marital status	Single	106 (24.4%)	220 (50.6%)	109 (25.1%)	0.009
	Married	46 (35.7%)	64 (49.6%)	19 (14.7%)	
Type of family	Nuclear family	70 (25.9%)	132 (48.9%)	68 (25.2%)	0.399
	Extended	82 (27.9%)	152 (51.7%)	60 (20.4%)	
Living area	Urban	115 (26.8%)	212 (49.4%)	102 (23.8%)	0.535
	Rural	37 (27.4%)	72 (53.3%)	26 (19.3%)	
Living place	Home	116 (27.8%)	206 (49.3%)	96 (23.0%)	0.669
	Dormitory	36 (24.7%)	78 (53.4%)	32 (21.9%)	
University	Herat University	110 (25.5%)	214 (49.7%)	107 (24.8%)	0.074
	Jami University	42 (31.6%)	70 (52.6%)	21 (15.8%)	
Year of study	First year	50 (27.9%)	83 (46.4%)	46 (25.7%)	0.561
	Second year	41 (27.9%)	72 (49.0%)	34 (23.1%)	
	Third and above	61 (25.6%)	129 (54.2%)	48 (20.2%)	
Faculty	Nonmedical	110 (26.0%)	210 (49.6%)	103 (24.3%)	0.250
	Medical	42 (29.8%)	74 (52.5%)	25 (17.7%)	
Father's education	Illiterate	48 (31.6%)	79 (52.0%)	25 (16.4%)	0.204
	School degree	46 (23.8%)	96 (49.7%)	51 (26.4%)	
	Bachelor and higher	58 (26.5%)	109 (49.8%)	52 (23.7%)	
Mother's education	Illiterate	85 (29.7%)	147 (51.4%)	54 (18.9%)	0.017
	School degree	41 (21.7%)	103 (54.5%)	45 (23.8%)	
	Bachelor and higher	26 (29.2%)	34 (38.2%)	29 (32.6%)	
Family income	High	34 (20.5%)	89 (53.6%)	43 (25.9%)	0.219
	Medium	86 (30.5%)	135 (47.9%)	61 (21.6%)	
	Low	32 (27.6%)	60 (51.7%)	24 (20.7%)	

## 4 Discussion

This study estimated the prevalence of IGD among students at Herat University and Jami University and examined its sociodemographic correlations. A high burden of IGD was observed—22.7% disordered, 50.4% at‐risk, and 27.0% normal (IGD‐20 categories)—providing a data‐driven basis for interpreting between‐group differences in the subsequent analysis.

When compared with previous studies, the prevalence observed in the present study was notably higher than most international estimates. For instance, rates reported in Saudi Arabia (21.5%) were comparable, whereas substantially lower rates were found among university students in Egypt (6.0%), Korea (5.9%), and India (approximately 5%–6%), including recent evidence among Indian medical students demonstrating significant associations between gaming disorder, psychological distress, and academic impact [15, 21, 22, 23, 24]. Similarly, studies conducted in China (10.3%) and during the COVID‐19 pandemic (9.9%) also reported lower prevalence than the current study [25, 26].

Such variation in reported IGD prevalence across regions may reflect methodological differences—particularly in diagnostic instruments and cutoff thresholds—as well as sociocultural and contextual disparities in gaming behavior and access to digital media [21, 23, 26, 27]. Furthermore, the considerably higher IGD prevalence in Herat may also relate to limited recreational opportunities, high internet engagement, and socioeconomic stressors faced by youth in post‐conflict Afghanistan. Additionally, the use of the IGD‐20 with relatively sensitive cutoff thresholds, reliance on self‐reported measures, increased post‐pandemic digital engagement, and the exclusive focus on university students may have contributed to elevated prevalence estimates compared with community‐based studies.

Multiple methodological and contextual factors can explain the observed differences in IGD prevalence across studies. Variation in assessment instruments, diagnostic criteria, and cutoff scores often leads to inconsistent prevalence estimates [21, 23, 26]. Beyond measurement issues, sociocultural norms, family supervision, and national attitudes toward gaming may also shape behavioral patterns [27, 28]. Differences in participants’ age, academic field, and study workload further contribute to the observed heterogeneity [15, 23, 29, 30].

Contrary to findings from several studies [27, 31] indicating a higher risk of IGD among students living away from home, the present study found a higher prevalence of IGD among those residing with their families. This contrast may reflect contextual differences in parental monitoring, family stress, or home‐based accessibility to smartphones and internet services. Moreover, the higher IGD rates observed among participants whose mothers had lower educational levels contrast with findings from India [23], where maternal education appeared protective. These differences may be shaped by broader gender and educational disparities in Afghanistan, where lower maternal education levels could limit awareness of healthy technology use and reduce parental control over gaming behaviors.

Consistent with earlier research from India, China, and Lebanon [23, 25, 27], previous evidence indicates that males are generally more susceptible to IGD than females. However, this pattern could not be examined in the present study due to the exclusion of female students, a limitation stemming from the current educational restrictions in Afghanistan.

Marital status was another significant predictor of IGD. Single participants demonstrated higher IGD levels, aligning with findings from Malaysia [26], where unmarried students showed greater gaming engagement. In contrast, studies from China [25] and India [23] did not report this relationship, suggesting that the influence of marital status on gaming behavior may depend on cultural norms, social support structures, and stress coping strategies.

Regarding game preferences, the most frequently played titles—PUBG, Clash of Clans, and Call of Duty—are highly competitive, multiplayer‐focused. Such games rely on continuous reinforcement mechanisms and social competition, which are known contributors to problematic gaming behavior [23, 26]. Their immersive design and constant reward feedback loops may explain the strong association between these game types and IGD symptoms observed among participants.

Beyond structural game characteristics, cognitive mechanisms appear to play an important role in the development and persistence of IGD. Maladaptive cognitions, such as persistent rumination about gaming, overvaluation of in‐game achievements, and distorted beliefs related to control, reward, or escape, have been shown to reinforce compulsive gaming behavior and impair self‐regulation. These cognitive processes may interact with environmental stressors and limited recreational opportunities, thereby amplifying the risk of IGD among university students in low‐resource and post‐conflict settings [8].

Although the IGD‐20 Test was used in the present study for its comprehensive coverage of DSM‐5 diagnostic domains, its length may limit its feasibility in large‐scale or time‐constrained surveys. Shorter validated instruments—such as the IGD scale–short form (IGDS9‐SF) and the gaming disorder test (GDT)—have demonstrated robust psychometric properties across diverse cultural and university‐based populations and may offer efficient alternatives for future epidemiological and clinical research [32, 33, 34].

This study has several limitations that should be acknowledged. First, only male participants were included due to the ongoing educational restrictions on female students in Afghanistan [35], which limits the generalizability of the findings across genders. Second, as data were obtained through self‐administered questionnaires, response bias and potential underreporting or overreporting of gaming behavior cannot be ruled out. Third, the cross‐sectional design precludes causal inference about the relationship between sociodemographic factors and IGD symptoms.

Moreover, this study did not assess additional psychological or academic variables—such as anxiety, depression, and academic performance—that may mediate or moderate IGD risk. Future research should incorporate both male and female participants, employ longitudinal designs, and include validated psychological scales better to understand the temporal and causal pathways of IGD. Interventions focusing on awareness campaigns, parental guidance, and campus‐based digital wellness programs are also recommended to mitigate the growing burden of IGD among university students in Afghanistan.

## 5 Conclusion

IGD is common among university students in Herat, with 22.7% disordered and 50.4% at risk. Only marital status and mother's education were significantly associated with IGD levels (*p* = 0.009 and *p* = 0.017, respectively); the maternal‐education pattern was non‐monotonic (higher disordered proportion in the bachelor's or higher group and lower at‐risk), indicating a mixed association rather than a simple gradient. Universities should implement screening, digital‐wellness education, and targeted support; future gender‐inclusive longitudinal studies are needed.

## Author Contributions

Mohammad Shafi Saljuqi, Abdul Wahid Hamidi, Mohammad Masudi, Ali Rahimi, Zalmay Majidi, Shafiq Ahmad Joya, and Nasar Ahmad Shayan conceptualized the manuscript. Mohammad Shafi Saljuqi, Abdul Wahid Hamidi, Mohammad Masudi, Ali Rahimi, Zalmay Majidi, Shafiq Ahmad Joya, and Nasar Ahmad Shayan wrote the original draft. Mohammad Shafi Saljuqi, Abdul Wahid Hamidi, Mohammad Masudi, Ali Rahimi, Zalmay Majidi, Shafiq Ahmad Joya, and Nasar Ahmad Shayan supervised the process. Mohammad Shafi Saljuqi, Abdul Wahid Hamidi, Mohammad Masudi, Ali Rahimi, Zalmay Majidi, Shafiq Ahmad Joya, and Nasar Ahmad Shayan reviewed and edited the manuscript.

## Funding

The authors have nothing to report. However, Jami University generously assisted with the costs of data collection, particularly the printing of questionnaires.

## Consent

The authors have nothing to report.

## Conflicts of Interest

The authors declare no conflicts of interest.

## Data Availability

The datasets generated and/or analyzed during the current study are available from the corresponding author, Dr. Mohammad Masudi (mhmasoudy313@gmail.com), upon reasonable request.
